# Children's Daily Routine Response to COVID-19 Emergency Measures in Serbia

**DOI:** 10.3389/fped.2021.656813

**Published:** 2021-04-20

**Authors:** Jovan Vuković, Radenko M. Matić, Ivana M. Milovanović, Nebojša Maksimović, Dragan Krivokapić, Saša Pišot

**Affiliations:** ^1^Faculty of Sport and Physical Education, University of Novi Sad, Novi Sad, Serbia; ^2^Faculty for Sport and Physical Education, University of Montenegro, Nikšić, Montenegro; ^3^Institute for Kinesiology Research, Science and Research Centre Koper, Koper, Slovenia

**Keywords:** children, school achievement, physical activity, screen time, COVID-19 pandemic emergency measures

## Abstract

**Objective:** The emergence of coronavirus in Serbia as well as in other European countries led to the declaration of a state of emergency, which, among other measures, included a switch to online education, the lockdown of public life and organized sports, and a curfew from 5 pm to 5 am. This study aimed to investigate the extent to which these measures affected children's daily routines. More specifically, it aimed to determine how children maintained their learning, physical activity, and screen time routines from the period before the state of emergency was declared.

**Methods:** Response to an online parent-reported questionnaire was conducted (*N* = 450). The factorial validity of the scales was prepared using confirmatory factor analysis, with acceptable fit indices. Based on that, the authors tested the interrelations between dimensions using structural equation modeling in SPSS, AMOS 24.0.

**Results:** The study results indicate a positive relationship between school achievement and study time (*β* = 0.25). They also indicate that children who were physically active before the pandemic continued their activities during the emergency state (*β* = 0.53). Physical activity impact during the COVID-19 emergency measures reduces children's behavior changes (*β* = 0.55). Finally, they highlight that children who spent more time with multimedia content had greater changes in anxiety, sensitivity, nervousness, and worry due to COVID-19 emergency measures (*β* = −0.38).

**Conclusions:** Healthy lifestyle habits formed in childhood are suggested to be responsible for the greater “resistance to change” shown by the children from this study.

## Introduction

As the pandemic Coronavirus Infectious Disease 2019 (COVID-19) has now been going on for a year, the results of numerous scientific studies suggest that the virus has left a major impact on the psychophysical health as well as on the social life of children and adults (1–3), namely, by the end of May 2020, 2.5 months after the pandemic was declared on March 11, 2020, (4), COVID-19 was already present in 188 countries, with 5,604,461 infected and 350,752 dead (5).

The first case of a COVID-19-positive patient in Serbia was reported on March 6, 2020. Following the example of other European countries and in the absence of other effective epidemiological measures, state authorities declared a state of emergency on March 15, 2020 to keep people at home, minimizing physical contact. These measures in Serbia included closing kindergartens, schools, and universities and switching to online teaching; recommending that the working population works from home; closing of parks, sports, and fitness centers to minimize physical contact. People over 65 years old were completely banned from leaving their homes, and for all other age groups, the curfew applied on March 16, 2020 was from 8 pm to 5 am and then on March 22, 2020 to April 30, 2020 from 5 pm to 5 am. According to official statistical data, by the end of May 2020, 11,275 positive cases for COVID-19 were registered in Serbia, with a total number of 240 deaths (6).

Concerning the fact that the focus of this research is on children aged 7–15 years, the epidemiological measures mentioned included a sudden transition to online education as well as a reduction in physical contact and finally a reduction of organized physical activity (PA) and free play outside houses and apartments. The results of previously published studies suggest that school-age children are at a higher risk after a long period without school commitments, especially in terms of nutrition, PA, motor skills, and, finally, general psychophysical health (5, 7, 8). More negative emergency epidemiological measure consequences were confirmed by the US research findings of significant decreases in physical activity, increases in sedentary behavior, and disrupted sleep schedules/sleep quality in children and adolescents (9). Certain differences of PA level have also been shown in a survey of US children where parents of older children (ages 9–13) *vs*. younger children (ages 5–8) perceived greater decreases in PA and greater increases in sedentary behavior in comparison to pre- to early-COVID-19 emergency measure (EM) periods (10). A Slovenian study also raises concerns about the consequences of EM time without school PA and sports. Data from more than 20,000 children from 1st to 9th grade of primary schools showed alarming trends of change: a decline in motor skills in both boys and girls, with overall motor performance decreased on average by more than 13%. The greatest decline occurred in endurance and in whole-body coordination between the last measurements in April 2019 and the measurements after the epidemic status in June 2020 (11). The above-mentioned changes of children's behavior will develop into long-term poor health outcomes in children and adolescents (12). With this in mind, we were interested in the level and type of daily PA of children attending “school from home” under exceptional conditions (the state of emergency, with emphasis on restricted freedom of movement).

For this purpose, we create a questionnaire on PA of children during the state of emergency caused by the COVID-19 pandemic. The survey was conducted in the Republic of Serbia, in the autonomous province of Vojvodina territory.

**Figure 1:**
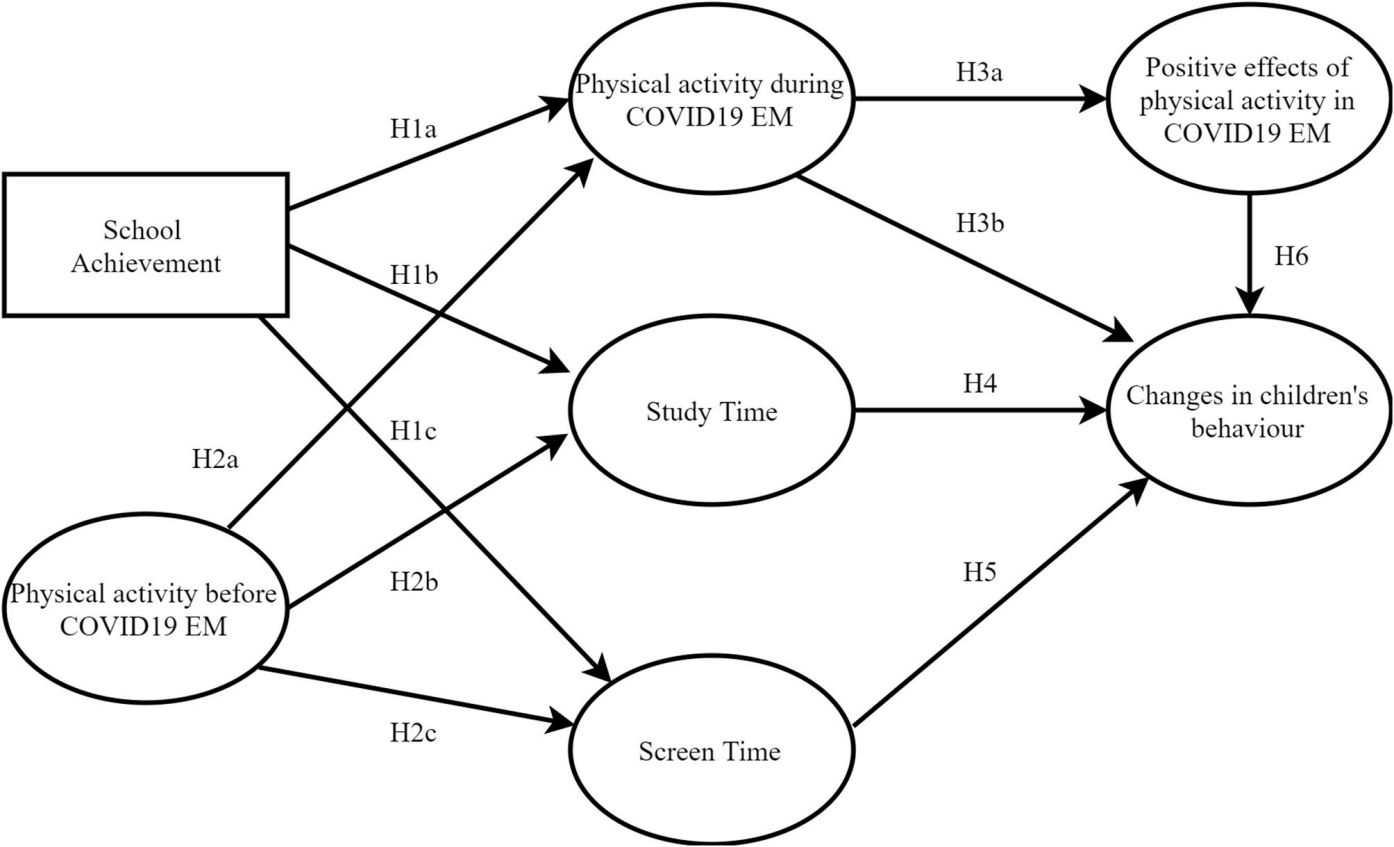
Proposed hypothesized model.

Based on the new “externally imposed” framework of children's daily life, we decided to analyze several key parameters of their lives, namely, we conducted the analysis of interrelation of children's PA before and during the pandemic COVID-19 within the variables: school achievement, study time, and screen time. For the purpose of this study, we have chosen these “pillars” of children's daily lives because they are key parameters whose continuity could be maintained, reduced, or increased during EM. The above-mentioned literature review considered that children's time during the EM could influence their behavior changes. Thus, we expected that factors that describe part of the children's daily routines before COVID-19 EM (such as school achievement and PA) could influence their daily routine during EM (PA, study time, and screen time). The examination of these interrelations was defined in hypotheses H1a, H1b, H1c, H2a, H2b, and H2c (see Figure 1). Furthermore, we expected that spending time during EM by children could influence the tested model's dependent variable—changes in children's behavior (hypotheses H3a, H3b, H4, H5, and H6 in Figure 1).

## Materials and Methods

### Data Collection

The online parent-reported questionnaire was conducted after the cancelation of the state of emergency in Serbia. According to a convenient way of collecting questionnaires that did not endanger the respondents' safety, the dissemination was conducted *via* schools, with the mediation of an association of physical education teachers who supervised the data collection. At the beginning of the online survey, all participants were informed about the research purpose. It lasted ~10 min, with protection of the anonymity of the respondents. The field research was conducted between May 29 to June 6, 2020.

### Study Sample

The survey included parents of children (228 males and 222 females, *N* = 450) from primary schools from the territory of Vojvodina, Serbia. The parent sample included 165 respondents (36.7%) whose children belong to younger school-age and 285 respondents (63.3%) with children of older school-age. By developing the research strategy, we decided that the online questionnaire should be completed by the parents, namely, we considered that the scale of parental assessment of the children's daily activities during COVID-19 EM would be more valid than the children's answers for two main reasons: this would prevent a possible misunderstanding of some questions at a younger age (7–10) or giving socially desirable answers at an older age (11–13, 13–15). The respondents' type of housing showed that 25.1% of them have apartments and houses without a yard, and 74.9% have houses with a yard. Furthermore, related to the type of settlement, the respondent sample consisted of respondents from the countryside (24.6%), suburbs (17.4%), and urban area (58.0%).

### Measures/Variables

The average grade measured the child's school achievement at the end of the previous grade. For this variable, an item that provided a score for academic efficiency was used, where score two means sufficient achievement, 3—good, 4—very good, and 5—excellent.

Three items measured PA before COVID-19 EM: (1) children's previous participation in an organized way of PA (three times a week) as members of sports clubs (yes or no), (2) previous regular sports and recreational activity or a combination of several different recreational activities such as aerobics, jogging, dancing, gymnastics, basketball, swimming, *etc*., (yes or no), and (3) children's involvement in any sports and recreational exercises in a sports club, outdoor sports, and exercise at home (in hours).

Physical activity during COVID-19 EM was measured with three items. The first item provides information about the children's PA at home (yes or no). Furthermore, this dimension includes items related to the amount of time the child spends in exercising at home (in minutes) and overall status of the children's physical form with exercise at home compared to the pre-quarantine period, with responses on five-point Likert scales (e.g., 1 = in the lowest level to 5 = completely as before). It should be emphasized that online school sports for children in primary school were performed *via* TV shows, and athletic training for children in sports clubs *was* via remote platforms (with a delay due to adaptation to the new circumstances).

Study time was measured with three items (e.g., how much time per day that your child spent on reading books, taking online classes, and learning or searching for information needed for class assignments in the last 2 months).

Screen time was measured by including four items that summarize information about the time (in hours) the child spent in using various multimedia sources: (1) single or multiplayer computer games, (2) video and music, (3) TV, and (4) social networking.

The positive effects of PA in COVID-19 EM were measured with three items related to the parents' opinions on the impact on their child's concentration, motivation, and good mood (e.g., I notice that my child has, after exercising at home, better concentration, motivation, *etc*.).

Changes in children's behavior were assessed with four items related to the parents' perceptions of various statements regarding their children's worry, anxiety, sensitiveness, and nervousness during quarantine (e.g., I notice that my child is more sensitive, worried, anxious, and nervous than before).

### Data Analysis

Statistical procedures included structural equation modeling (SEM), conducted using SPSS, IBM, and AMOS 24.0, which tested the relationships of a hypothesized model of constructs. This method was applied as a multivariate, complex method that included factor and multiple regression analysis.

At the beginning of the statistical procedure, the SEM measurement model's reliability and validity were applied. Reliability was tested using Cronbach's alpha, considering the thresholds for *α* as proposed by Kline (14), according to which excellent values are *α* ≥ 0.90, good values were between 0.80 and 0.90, and values between 0.70 and 0.80 are considered acceptable.

The SEM measurement model's validity was realized using the factor loadings and the average variance extracted (AVE). An accepted value of 0.50 is suggested as a minimum threshold for AVE (15, 16). The root mean square error of approximation (RMSEA), comparative fit index (CFI), Tucker–Lewis index (TLI), and non-normed fit index (NNFI) have been calculated as model fit indices. According to Byrne (17) and Steiger (18), the value of RMSEA shows a good fit in the range from 0.05 to 0.10, and a “good fit” for CFI, TLI, and NNFI “was >0.95 (18, 19). Otherwise, the values of CFI, TLI, and NNFI ≥0.90 can be recognized as an acceptable fit.

In this model, school achievement and PA before COVID-19 EM were set as independent variables, three dimensions (physical activity during COVID-19 EM, study, and screen time) were mediator variables, and changes in children's behavior were set as a dependent variable. Mediation analysis (20, 21) was conducted using the Sobel test (*p* < 0.001).

## Results

The obtained results were divided into three segments: (1) descriptive statistics, (2) confirmatory factor analysis, and (3) structural equation modeling. According to the descriptive statistics (Table 1) of school achievement, the sample consisted of mostly excellent (65.4%), very good (27.3%), some less good (6.2%), and sufficient pupils (1.2%). The status of children's PA before COVID-19 EM considered that 70.2% of children were in sports clubs three times per week. Concurrently, other variables in this dimension showed a high level of PA (about 75% participated in a combination of different sport recreation activities three times per week, and only 12.2% of children had <0.5 h in sport recreation activity per day).

**Table 1:** Descriptive statistics of children's school achievement and physical activity before COVID-19 emergency measures (EM) and children's daily routine during COVID19 EM.

	**Total**	**Male**	**Female**
**School achievement (%)**			
Sufficient	1.2	1.8	0.5
Good	6.2	7.7	5.2
Very good	27.3	31.4	23.0
Excellent	65.4	59.1	71.8
**Physical activity before COVID19 EM (%)**			
**Member of sports clubs**			
Yes	70.2	71.1	69.4
No	29.8	28.9	30.6
**Combination of different sports recreation activity of children**			
Yes	74.8	75.0	74.7
No	25.2	25.0	25.3
**Average in sports recreational activity per day**			
<1/2 h	12.2	14.1	9.8
<1 h	29.3	30.0	29.4
<2 h	30.9	30.8	30.4
<3 h	12.4	11.9	13.1
>3 h	11.1	11.0	11.2
Do not know	4.0	2.2	6.1
**Physical activity during COVID19 EM**			
**Exercise at home (%)**			
Yes	63.4	64.2	63.4
No	36.6	35.8	36.6
**Assessment of the measure of maintaining physical form (%)**			
Partially	69.0	64.5	73.6
Satisfactorily	24.4	29.5	18.9
Complete	6.6	6.0	7.5
Exercise at home (min)			
<10 min	18.1	15.4	19.2
<30 min	50.0	55.2	44.9
≥60 min	31.9	29.4	35.9
**Study time**			
**Reading books**			
<1 h	25.0	28.3	21.2
<2 h	29.5	33.6	25.0
≥3 h	45.5	38.1	53.8
**Online teaching, learning per day**			
<1 h	16.0	12.6	19.8
<2 h	33.3	31.8	33.5
≥3 h	51.7	55.6	46.7
**Searching information for teaching on the Internet (hours)**			
<2 h	83.3	83.6	84.0
2–4 h	12.7	13.3	11.7
4–6 h	4.0	3.1	4.2
**Screen time**			
**Single and multiplayer computer games**			
<2 h	84.1	84.4	83.5
2–4 h	12.3	11.1	13.7
4–6 h	3.6	4.4	2.8
**Social networks (%)**			
<2 h	65.0	65.5	63.8
2–4 h	22.3	19.0	26.3
4–6 h	12.7	15.5	9.9
**Video, music**			
<2 h	87.6	89.7	84.8
2–4 h	10.1	7.6	13.3
4–6 h	2.2	2.7	1.9
**Television**			
<2 h	79.5	80.9	78.4
2–4 h	19.2	17.3	21.1
4–6 h	1.3	1.8	0.5
Positive effects of physical activity	Yes	No	Yes
Concentration	65.1	34.9	64.8
Motivation	63.7	36.3	65.3
Attention	55.6	44.4	54.5
**Changes in children's behavior**			
Sensitivity	26.4	73.6	25.0
Worry	29.8	70.2	29.4
Anxiety	38.3	61.7	34.9
Nervousness	25.5	74.5	26.4

Table 1 also shows the descriptive statistics of children's daily routine during COVID-19 EM. A comparison of PA before and during COVID-19 EM revealed a small decrease in the number of PA participants. Thus, it is visible that 70.2% of children were a member of sports clubs before COVID-19 EM compared to 63.4% of children who exercise at home during the pandemic. That difference was confirmed in another dimension of the PA variables during COVID-19 EM, where 69% of parents considered PA level only enough to maintain physical form partially. Almost two-thirds of children exercised at home for <30 min.

An evaluation of screen time showed that children spent more hours using different screen devices than the American Academy of Pediatrics' recommendations. These recommendations considered 1 up to 1.5 h per day for elementary school-aged children and up to 2 h per day for middle school-aged children (22).

The positive effects of the PA were revealed on the concentration (65.1%), motivation (63.7%), and attention (55.6%) of children. Otherwise, every fourth child felt more sensitive, worried, and nervous than before COVID-19 EM.

The validity and reliability measures are calculated in Table 2. Firstly, it is considered that Cronbach's alpha values were acceptable (>.80), which is suggested by Kline (14). Subsequently, AVE values fulfill the criteria >0.50 as proposed by Fornell and Larcker (15), ranging from 0.55 to 0.72.

**Table 2:** Descriptive statistics and CFA item statistics and validity and reliability indices of the measurement model.

**Constructs**	**Items**	**Descriptive statistics**				**Standardized factor loadings (*****p*** **< 0.001)**		**Validity and reliability measures**		
		**M**	**SD**	**SK**	**KT**	**Factor loading**	**SE**	**SMCs**	***α***	**AVE**
School achievement	SA1	4.57	0.66	−1.49	1.85					
Physical activity (PA) before C19 Quarantine	PAB1	1.70	0.45	−0.89	−1.22	0.51	0.02	0.26	0.81	0.55
	PAB2	1.74	0.45	−1.32	0.32	0.91	0.07	0.83		
	PAB3	3.63	1.34	0.11	−0.60	0.90	0.12	0.01		
PA during C19 quarantine	PAD1	2.47	0.54	−0.28	−1.12	0.51	0.01	0.39	0.80	0.61
	PAD2	3.77	1.22	−0.06	−0.86	0.91	0.11	0.83		
	PAD3	3.82	1.34	−0.18	−0.66	0.58	0.11	0.34		
Study time	LT1	5.08	1.09	−1.23	1.20	0.65	0.07	0.01	0.90	0.72
	LT2	2.72	0.89	0.48	0.12	0.55	0.10	0.30		
	LT3	2.26	0.85	0.69	0.68	0.86	0.21	0.74		
Screen time	ST1	2.80	1.38	0.03	−1.26	0.57	0.13	0.32	0.91	0.68
	ST2	2.29	1.03	0.46	−0.42	0.45	0.06	0.20		
	ST3	2.12	1.18	0.74	−0.55	0.56	0.09	0.31		
Positive effects	PE1	1.65	0.48	−0.63	−1.61	0.80	0.01	0.63	0.90	0.70
	PE2	1.64	0.48	−0.57	−1.68	0.77	0.01	0.59		
	PE3	1.56	0.50	−0.22	−1.96	0.66	0.01	0.44		
Changes in children's behavior^#^	CB1	1.73	0.44	−1.04	−0.93	0.55	0.01	0.05	0.81	0.59
	CB2	1.86	0.35	−2.05	2.22	0.55	0.01	0.30		
	CB3	1.54	0.50	−0.14	−1.99	0.54	0.01	0.29		

*M, mean; SD, standard deviation; SK, skewness; KT, kurtosis; SE, standard error; SMCs, squared multiple correlations; α, Cronbach's alpha; AVE, average variance extracted*.

# *A variable with inverse metrics*.

The measurement model was shown to be with acceptable fit: χ^2^ = 2,130.6, *df* = 530, χ^2^/*df* = 4.02, CFI = 0.91, TLI = 091, NNFI = 0.90, and RMSEA = 0.05. Therefore, the values of CFI, TLI, and NNFI confirmed the acceptable fitting of the model suggested by Byrne (17) and Hu and Bentler (19). RMSEA of 0.05 also satisfied the criteria proposed by Byrne (17), and Steiger (18) fits within the range from 0.05 to 0.10. All calculated factor loadings are more than 0.4, which is a suggestion by Kaiser (23). Their minimum value is 0.45, which means that the applied measurement model fits well with the empirical data.

**Figure 2:**
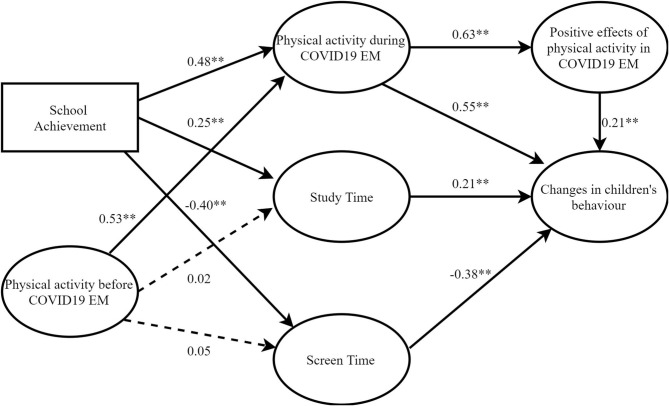
The results of the proposed hypothesized model - Direct and Indirect Impact of School Achievement, Physical Activity before COVID19 EM, Physical Activity during COVID19 EM, Study Time and Screen Time on Changes in children's behavior.

In Figure 2, the path analysis showed that the applied model explains 47% of the variance of the criterion variable changes in children's behavior, suggesting a further search for additional variables whose inclusion in the model would increase this percentage of variance explanation (to 80% and more).

The variable physical activity of the child before COVID-19 EM had a positive indirect effect through the variable physical activity during the COVID-19 EM on the dependent variable changes in children's behavior. It shows that physically active children had less anxiety, worry, nervousness, and sensitive behavior during the pandemic. This finding is consistent with previous research indicating that PA reduces, among other things, anxiety and the risk of obesity and early metabolic risk factors (24–27). Additionally, during childhood, PA levels tend to impact adolescence and adulthood, which is another reason for regular PA in childhood (24, 28).

School achievement directly affected the variable study time, showing that better students spent more time studying or searching for study materials during the EM. Such a result shows that children's study habits are resistant to changes in the daily routine.

During COVID-19 EM, PA showed a direct effect on reducing changes in the variable changes in children's behavior, suggesting that physically active children had fewer behavior changes during quarantine than children who were not physically active. This result also suggests that positive/healthy routines formed in early childhood are more resistant to change. Such results (school achievement and PA during COVID-19 EM) are significant, especially if we consider that the closure of schools, distance learning, and minimization of physical contact with other people is the first state of emergency in the lives of Vojvodina children.

The variable screen time showed a negative direct impact on the variable changes in children's behavior, suggesting that children who spent more time with multimedia content showed greater changes in anxiety, worry, sensitivity, and nervousness to COVID-19 EM. The findings are consistent with previous research indicating a linear association between total screen time and less acceptable temperament dispositions, degraded socio-emotional relations, and decline of health-related indicators of quality of life (29).

The variable study time showed a direct effect on changes in children's behavior, which means that more time focused the children's attention on learning materials, which contributed to reducing changes in the mentioned characteristics of children's behavior.

## Discussion

This research represents a contribution to the study and understanding of school-age children's response and adaptation to the EM caused by the first wave of COVID-19 pandemic in Spring 2020. The research covered several “pillars” of children's daily lives: school achievement, study time, PA (before and during the pandemic), and screen time during the pandemic.

### School Achievement and Study Time

A positive correlation between school achievement and study time was confirmed (29), which is consistent with spending more time studying or searching for study materials by better students during the EM. Additionally, it showed that the children's study habits are more resistant to changes in the daily routine. This result indicates the importance of establishing healthy lifestyle and learning habits already in early childhood. Sociologically speaking, the dispositions for action—the “habitus” of a family that carries acceptable action patterns for the family and society—will, based on the practices of action, design a “healthy” or “unhealthy” lifestyle. Parents are the ones who form permanent dispositions for future behavior, namely, as family practices through the child's perceptions (experiences) are also considered as appropriate by the society as well as within the possibilities (material resources), attitudes, and behaviors (30) and form a “healthy family lifestyle.”

### PA (Before and During the Pandemic)

The results of the study show the continuation of PA routine at children who were physically active before the pandemic and consequently showed fewer negative behavioral changes such as anxiety, fear, quarrelsome behavior, indifference, and laziness developed due to COVID-19 EM in contrast to children who spent more time with multimedia content and were less physically active and sedentary. Although the recommendations for regular PA of children basically refer to living in “ordinary” social circumstances, the results of this research indicate that their importance became even more obvious during COVID-19 EM. The limited freedom of outdoor movement and the prolonged stay in the house/apartment are circumstances for development of risk of new inactive lifestyle habits.

### Screen Time During the COVID-19 Pandemic

A negative direct impact of screen time on the variable changes in children's behavior suggests negative and greater changes in children who spent more time with multimedia content as shown in their anxiety, worry, sensitivity, and nervousness to COVID-19 EM. These results are consistent with previously conducted research (31), pointing to the fact that the long-term exposure of children to screens (watching television and movies) leads to changes in children's behavior that manifest themselves in the area of social problems, thinking problems, and aggressive behavior as well as social withdrawal and social isolation (in the case of video games). In addition, the more time that children spend in front of screens, the less time they have for all other daily activities. The consequences of such daily activities in childhood are directly reflected in lifestyles at later stages of adolescent development.

## Conclusion

The research findings suggest that healthy lifestyle habits formed in childhood, emphasizing regular PA and study habits, are responsible for the greater “resistance to change” of the children from this study. Considering that this was the first epidemiological EM life experience for the studied population, the results' significance is more obvious. Additionally, this result is compatible with a similar research conducted on Italian adolescent samples (2). The results showed that adolescents manifested the ability to live and adapt to the new, insecure life situation, which led them to the “new normal” daily life.

Finally, our results related to screen time are consistent with the findings of previous studies on children and youth populations (31–33). Screen time and sedentary behaviors are features of people's lifestyles in contemporary societies (34–37). However, despite some limitations of the study, this research indicates the need to emphasize the impact of lockdown and limited freedom of movement on behavioral or daily routine changes in line with the health-promoting recommendation of balanced PA and sedentary behavior in children and adolescents.

A brief research report has study limitations, which can be marked as significant for directions in similar future research. There were independent variables selected in the theoretical model, school achievement, and PA as important indicators of the children's previous daily routines before COVID-19 EM. This model does not include the local community's effect on its pandemic strategies and actions on the ground. Regarding the pandemic situation, the environment for children's daily routine functioning and manifestation could be different. The sample included children from the whole administrative province of Vojvodina, where some pandemic measures were different during the pandemic period.

Additionally, the model could be developed with more variables for examining children's active or passive lifestyle before the beginning of the pandemic. Therefore, this model can be tested in different contexts (e.g., other destinations, additional lifestyle dimensions, *etc*.). Finally, it was cross-sectional research, which means that there is a lack of detailed monitoring of changes in children's behavior.

However, this type of research can provide valuable information about how children's daily routines respond to unforeseen circumstances. This brief report's results can provide insight for the academic community and policymakers on what healthy life habits are essential for children's daily routine in potentially similar social circumstances. Finally, since the family and the school are important agents of children's socialization, the role of parents and teachers in promoting and maintaining the above-mentioned healthy lifestyle habits of children is of key importance. Therefore, it is important to inform them on the results of this and similar research. By warning and empowering both classroom teachers and parents about the importance of healthy lifestyle education, the negative consequences of similar EM can be mitigated in the future.

## Data Availability Statement

The raw data supporting the conclusions of this article will be made available by the authors, without undue reservation.

## Ethics Statement

The studies involving human participants were reviewed and approved by Ethical Committee of the Faculty of Sport and Physical Education, University of Novi Sad, Serbia (46-11-07/2020-1). Written informed consent for participation was not required for this study in accordance with the national legislation and the institutional requirements.

## Author Contributions

JV, RM, IM, and SP wrote the manuscript, performed analyses, and revised the manuscript. JV, RM, and DK collected the data. IM, RM, and NM overviewed previous studies and discussed the results. All authors contributed to the article and approved the submitted version.

## Conflict of Interest

The authors declare that the research was conducted in the absence of any commercial or financial relationships that could be construed as a potential conflict of interest.
